# Effect of β-lactam antibiotics on plant regeneration in carrot protoplast cultures

**DOI:** 10.1007/s11627-014-9626-0

**Published:** 2014-07-04

**Authors:** Ewa Grzebelus, Lukasz Skop

**Affiliations:** Institute of Plant Biology and Biotechnology, Faculty of Horticulture, University of Agriculture in Krakow, Al. 29 Listopada 54, 31-425 Krakow, Poland

**Keywords:** *Daucus carota*, Carbenicillin, Cefotaxime, Somatic embryogenesis, Timentin

## Abstract

Protoplasts of three carrot cultivars were isolated from *in vitro*-grown plantlets by overnight incubation in an enzyme mixture composed of 1% (*w/v*) cellulase Onozuka R-10 and 0.1% (*w/v*) pectolyase Y-23. After cell immobilization in modified thin alginate layers, three types of β-lactam antibiotics (cefotaxime, carbenicillin, or timentin) at five different concentrations (100, 200, 300, 400, or 500 mg L^−1^) were added to the culture medium. In 20-d-old cultures, a different number of cell colonies had formed and varied on average from 27 to 56% in carbenicillin- and cefotaxime-containing media, respectively. Supplementation of the culture media with antibiotics at concentrations higher than 100 mg L^−1^ resulted in a decrease in plating efficiency in comparison with the controls. However, from all antibiotic treatments, except carbenicillin at concentrations of 400–500 mg L^−1^, efficient plant regeneration occurred. For this reason, we believe that cefotaxime and timentin in the concentrations analyzed here may be used in complex *in vitro* procedures or valuable carrot cultures as a prophylactic agent for prevention against occasional contaminations.

## Introduction

Plant protoplast technology can be widely used in various aspects of plant science to study such fundamental processes as differentiation, dedifferentiation, and pluripotency of cells (reviewed in Jiang *et al.* 2013), and to introduce novel characteristics in commercial crops. Conventional breeding tools may incorporate different techniques exploring protoplast culture, including micropropagation, *in vitro* selection, genetic transformation, and, particularly, somatic hybridization (reviewed in Davey *et al.* 2005a). Systems based on protoplast culture contain many steps and include time-consuming procedures as follows: (1) induction of *in vitro*-grown donor material; (2) source tissue slicing, conditioning, and digestion; (3) protoplast isolation and purification; (4) for some species, embedding of the cells before culture; and finally, (5) plant regeneration preceded with stepwise reduction of the osmotic pressure by diluting the medium (Davey *et al.* 2005b). Microbial contamination caused by bacteria, yeasts, and fungi introduced at any of these steps can become a serious problem, especially when it occurs on rare, valuable, or irreplaceable cultures. As a result, a reduced growth rate is observed usually leading to cell/tissue/plant death, which eliminate the cultures completely from the program, and the long procedure of donor material establishment, protoplast isolation, or their regeneration into plants must be started again (Leifert and Waites 1992; Shehata *et al.* 2010).

Contamination introduced to *in vitro* cultures can be exogenous or endogenous originating from explant surfaces, intracellular spaces within the plant tissues, or poor aseptic conditions during manipulation in laminar flow hoods (Shehata *et al.* 2010). They may express themselves immediately or can remain latent for a long period of time, which makes them extremely difficult to control (Leifert and Cassells 2001). Contamination losses during the *in vitro* stages of plant tissue culture may be substantially reduced or eliminated by using antimicrobial treatments such as antibiotics (Abdi *et al.* 2008). Despite their common and successful application to minimize bacterial growth in animal cell culture, they are frequently phytotoxic and may differently affect the regeneration ability in plant cell and tissue culture (Pollock *et al.* 1983; Abdi *et al.* 2008). Antibiotics were shown either to retard/inhibit (da Silva and Fukai 2003; da Silva Mendes *et al.* 2009; Qin *et al.* 2011) or stimulate explant growth and development (Costa *et al.* 2000; Mittal *et al.* 2009; Shehata *et al.* 2010). Their role in affecting the developmental events is not well understood, but it has been assumed that the antibiotics mimic plant hormones since some of them possess an auxin-like structure (Grewal *et al.* 2006; Qin *et al.* 2011). Plant sensitivity to antibiotics usually is species-specific and mainly depends on growth conditions, explant type, and culture system (Qin *et al.* 2011). Therefore, before their application to the culture in order to prevent, minimize, or eliminate unwanted microorganisms, it is necessary to screen the type and concentration of antibiotics with the least phytotoxic effects on plant tissues and cells.

To suppress or eliminate gram-negative and/or gram-positive bacteria infecting *in vitro* cultures, antibiotics with a broad spectrum of microbiological activity and with little or no detrimental effect on plant growth and regeneration should be used (Cheng *et al.* 1998). β-Lactam antibiotics such as carbenicillin (belonging to the penicillin group [Holford and Newbury 1992]), cefotaxime (a semisynthetic analog of cephalosporin [Danilova and Dolgikh 2004]), and, recently, timentin (a mixture of a penicillin derivative ticarcillin and clavulanic acid [Nauerby *et al.* 1997]) are most commonly used to control bacterial growth in plant tissue culture particularly after *Agrobacterium*-mediated transformation (Tang *et al.* 2000; Ogawa and Mii 2004; da Silva Mendes *et al.* 2009; Qin *et al.* 2011). It is known that β-lactams interfering with penicillin-binding proteins in the bacterial periplasm inhibit the biosynthesis of peptidoglycan—a specific component of the prokaryotic cell wall that in consequence provokes bacterial death by cell wall lysis (Nauerby *et al.* 1997; Ogawa and Mii 2004). The effect of β-lactam antibiotics on the development of such sensitive structures as plant protoplasts has rarely been investigated, especially at high concentrations, which are usually required to control microbial infections in culture systems (Simmonds and Grainger 1993; Teng and Nicholson 1997). To our knowledge, there are only a few reports showing the influence of selected antibiotics on protoplast cultures of *Antirrhinum majus*, *Pisum sativum*, and *Nicotiana tabacum* (Watts and King 1973); *Nicotiana plumbaginifolia* (Pollock *et al.* 1983); *Passiflora edulis* (d’Utra Vaz *et al.* 1993); *Triticum aestivum* (Simmonds and Grainger 1993); and *Allium longicuspis* (Fellner 1995). It seems that carrot (*Daucus carota* L. ssp. *sativus* Hoffm., 2*n* = 2*x* = 18), a model species for plant tissue culture systems and one of the most important vegetable crops in Apiaceae family, has not been investigated in this context. Therefore, the objective of the present study was to compare the effects of three β-lactam antibiotics, carbenicillin, cefotaxime, and timentin, in different concentrations on plant regeneration capacity, in carrot protoplast cultures in order to define those which were less toxic to plant cells for prophylactic use against bacterial contamination in procedures based on protoplast isolation and culture.

## Materials and Methods

### Plant material.

Three open-pollinated carrot (*D. carota* L. ssp. *sativus* Hoffm.) cultivars were used as donors for protoplast isolation: ‘Dolanka’, ‘Amsterdamska’, and ‘Koral’ (all provided by Polan-Polish seed company). Protoplasts were isolated from *in vitro*-grown plantlets derived from seeds as described previously by Grzebelus *et al.* (2012). Briefly, seeds were germinated *in vitro* after surface sterilization with a three-step procedure including incubation (1) in a water bath at 40°C, then (2) in 0.2% (*v/v*) solution of fungicide ‘Bravo’ (Syngenta, Waterford, Ireland), and finally (3) in 20% (*w/v*) solution of chloramine T trihydrate (N-chloro-p-toluenesulfonamide sodium salt) 30 min each, followed by three rinses with sterile distilled water. After air-drying on a filter paper, the seeds were placed in Petri dishes on solid Murashige and Skoog (MS) medium with vitamins (Murashige and Skoog 1962) supplemented with 30 g L^−1^ sucrose and 6.5 g L^−1^ plant agar (Biocorp, Warszawa, Poland) and incubated at 26 ± 2°C in the dark. After approximately 1 wk of culture, seedlings were transferred to glass jars containing regeneration medium (R) composed of MS macro- and micro-elements, 0.1 mg L^−1^ thiamine HCl, 0.1 mg L^−1^ pyridoxine HCl, 0.5 mg L^−1^ nicotinic acid, 3.0 mg L^−1^ glycine, 100 mg L^−1^ myo-inositol, 20 g L^−1^ sucrose, and 2.5 g L^−1^ Phytagel (Sigma-Aldrich, St. Louis, MO, USA). Cultures were kept in a climate room at 26 ± 2°C under a 16-h photoperiod and light intensity of 55 μmol m^−2^ s^−1^.

### Isolation and culture of protoplasts.

Protoplasts were isolated and cultured according to a previously established protocol (Grzebelus *et al.* 2012). Briefly, approximately 1 g of leaves with petioles of 3- to 4-wk-old plantlets were sliced into small pieces in 0.5 M mannitol solution and incubated for 1 h. Enzymatic release of protoplasts took place overnight (14–16 h) on a rotary shaker (30 rpm) in a solution consisting of 1% (*w/v*) cellulase Onozuka R-10 (Duchefa Biochemie, Haarlem, The Netherlands), 0.1% (*w/v*) pectolyase Y-23 (Duchefa), 20 mM 2-(N-morpholino)ethanesulfonic acid (MES, Sigma), 5 mM CaCl_2_, and 0.6 M mannitol (Sigma), pH 5.6, filter-sterilized (0.22 μm; Millipore, Billerica, MA). Both plasmolysis of the source tissue before enzyme treatment and enzymatic digestion were conducted in the dark at 26 ± 2°C. Then, the mixture was sieved through an 80-μm nylon mesh (Millipore) and centrifuged at 100*g* for 5 min. The pellet was resuspended in 8 mL of 0.5 M sucrose with 1 mM MES and overlaid with 2 mL of W5 medium (Menczel *et al.* 1981). Following centrifugation at 145*g* for 10 min, intact protoplasts suspended at the solute gradient interface were collected and washed twice by resuspending in W5 solution and the culture medium, respectively, and centrifuged at 100*g* for 5 min after each wash. The working protoplast density was estimated using a Fuchs Rosenthal hemocytometer and adjusted to 8 × 10^5^ protoplasts per milliliter. Then, the protoplasts were immobilized in modified thin calcium alginate layers at a final plating density of 4 × 10^5^ mL^−1^ and cultured in the CPP medium consisting of macro-, micro-elements, and organic acids according to Kao and Michayluk (1975), vitamins according to B5 medium (Gamborg *et al.* 1968), 74 g L^−1^ glucose, 250 mg L^−1^ casein enzymatic hydrolysate (Sigma), 0.1 mg L^−1^ 2,4-dichlorophenoxyacetic acid (2,4-D), and 0.2 mg L^−1^ zeatin (pH 5.6, filter-sterilized). The cultures were incubated in the dark at 26 ± 2°C. The medium was replenished every 10 d.

### Antibiotics.

Three types of β-lactam antibiotics were used in the experiments: cefotaxime sodium (Polfa-Tarchomin S.A., Warszawa, Poland), carbenicillin disodium (Duchefa), and timentin (ticarcillin disodium/clavulanate potassium = 1,500/100; GlaxoSmithKline, London, UK). They were dissolved in double distilled water, filter-sterilized (0.22 μm, Millipore), and stored until use at −20°C. Working solutions of antibiotics were applied individually to the protoplast culture medium in five different concentrations: 100, 200, 300, 400, or 500 mg L^−1^. After 10 d of culture (simultaneously with refreshment of the medium), antibiotics were applied again.

### Plant regeneration.

All steps involving plant regeneration were the same as those presented by Grzebelus *et al.* (2012). Briefly, after 2 mo of culture in the dark at 26 ± 2°C, both proembryonic mass (PEM) and somatic embryos emerging from an alginate matrix in antibiotic-treated and control combinations were released from Ca-alginate layers by incubation in a sodium citrate solution. Following two rounds of centrifugation, the pellet finally consisted of callus and embryos free from alginate residue and citrate solution, and was carefully resuspended in the CPPD medium (1/4-strength macro-, micro-elements, and organic acids according to Kao and Michayluk [1975], vitamins according to B5 medium [Gamborg *et al.* 1968], 30 g L^−1^ sucrose, 20 g L^−1^ mannitol, and 250 mg L^−1^ casein enzymatic hydrolysate [Sigma], 0.1 mg L^−1^ NAA and 0.2 mg L^−1^ zeatin, pH 5.6) and plated on filter paper placed on the solidified R medium. Approximately 2–3 wk later, small-rooted plantlets were transferred to a fresh R medium for further growth. During plant regeneration, the cultures were maintained in a climate room at 26 ± 2°C under a 16-h photoperiod and light intensity of 55 μmol m^−2^ s^−1^.

### Data collection and statistical analysis.

To assess the effect of the selected antibiotics on protoplast growth, ability of the protoplast-derived cells to form aggregates was determined. For that purpose, 20 d after antibiotic application, plating efficiency was estimated and expressed as the number of cell aggregates per the total number of observed undivided cells and cell aggregates (×100). All microscopic observations were performed under an Axiovert S100 microscope (Carl Zeiss, Göttingen, Germany). During the regeneration step for each antibiotic-treated and control culture, the number of completely regenerated and normal plants per alginate layer was scored.

All experiments were carried out with at least two replications, each treatment being represented by three Petri dishes (for protoplast cultures) or at least three glass jars (for regeneration). For plating efficiency, counting was carried out in four microscopic fields on 200–600 cells per Petri dish. Mean values and standard errors were calculated. The overall effect of treatments was assessed using the analysis of variance (ANOVA) and Duncan’s honestly significant difference (HSD) test in Statistica 9.0 (StatSoft Inc. 2009). Additionally, for each accession, in order to estimate the relationships between concentrations of antibiotic and plating efficiency, analysis of linear regression was performed. The coefficient of determination (*R* ^2^) and Pearson’s correlation coefficient (*r*) were calculated to measure goodness of fit of a statistical model and the strength and direction of the linear relationship, respectively.

## Results

### Plating efficiency in the culture without antibiotics.

Around the third and fourth day of culture, some protoplasts had enlarged and started to change from a spherical to oval shape indicating a reconstruction of the cell wall, and in 5-d-old cultures, first mitotic divisions were observed. Cell divisions took place regularly, and in 20-d-old cultures, cell colonies of different sizes appeared with an average frequency of 52.4 ± 4.8% (Table 1). The plating efficiency varied from 42% for ‘Amsterdamska’ to 65% for ‘Dolanka.’ Even though the difference was marginally insignificant (*P* = .06).

**Table 1:** Plating efficiency of carrot donor accessions in a culture medium without antibiotics

Plating efficiency (% ± SE)			
Accession			Mean
Dolanka	Amsterdamska	Koral	
64.5 ± 7.4 a	41.8 ± 8.6 a	42.7 ± 4.7 a	52.4 ± 4.8

Means with the same *letters* were not significantly different at *P* ≤ .05

### Effect of antibiotics on plating efficiency.

The proportion of cell colony formation was highly dependent on the protoplast donor accession as well on the type and concentration of antibiotic (*P* ≤ .001). Twenty days after antibiotic application to the culture medium, the plating efficiency for ‘Dolanka’-derived protoplasts was approximately 1.5-fold higher than that for ‘Amsterdamska’ and ‘Koral’ reaching 50, 31, and 29%, respectively (Table 2). Of the antibiotics used, cefotaxime showed the least toxic effect on cell division frequency, while in the presence of carbenicillin and timentin, almost a twofold decrease was observed (*P* < .001; data not shown). Supplementation of the culture medium with antibiotic in the range of 200 to 500 mg L^−1^ reduced the plating efficiency in comparison to controls on average from 41 to 25%, respectively (Table 2).

**Table 2:** Average effect of type and concentration of antibiotics on plating efficiency in 20-d-old protoplast cultures of different carrot accessions

Factor	Plating efficiency (% ± SE)			Mean
	Antibiotic			
	Cefotaxime	Carbenicillin	Timentin	
Accession^a^				
Dolanka	62.6 ± 3.6 a	43.1 ± 7.0 a	37.9 ± 2.5 a	49.8 ± 3.4 a
Amsterdamska	62.7 ± 1.3 a	8.9 ± 2.3 b	22.7 ± 3.3 b	31.4 ± 4.1 b
Koral	36.7 ± 3.1 b	12.5 ± 4.3 b	36.8 ± 3.8 a	28.6 ± 2.9 b
Concentration of antibiotic^b^ (mg L^−1^)				
0	60.5 ± 7.3 a	50.1 ± 10.4 a	44.6 ± 4.7 a	52.4 ± 4.8 a
100	61.3 ± 5.9 a	40.3 ± 10.9 ab	36.0 ± 3.8 ab	46.7 ± 5.0 ab
200	57.7 ± 5.5 a	31.4 ± 10.8 abc	31.7 ± 5.2 ab	41.0 ± 5.2 bc
300	56.2 ± 6.3 a	22.7 ± 10.6 abc	30.6 ± 5.6 ab	37.1 ± 5.6 bc
400	53.0 ± 6.3 a	13.7 ± 9.4 bc	27.4 ± 5.3 ab	31.7 ± 5.6 cd
500	48.1 ± 6.9 a	3.0 ± 1.9 c	24.7 ± 5.2 b	25.3 ± 5.1 d

In each section of the table, means with the same *letters* did not differ significantly (*P* ≤ .001) within each *column*

^a^The means represent averages of all concentrations

^b^The means represent averages of the three accessions

**Figure 1.**
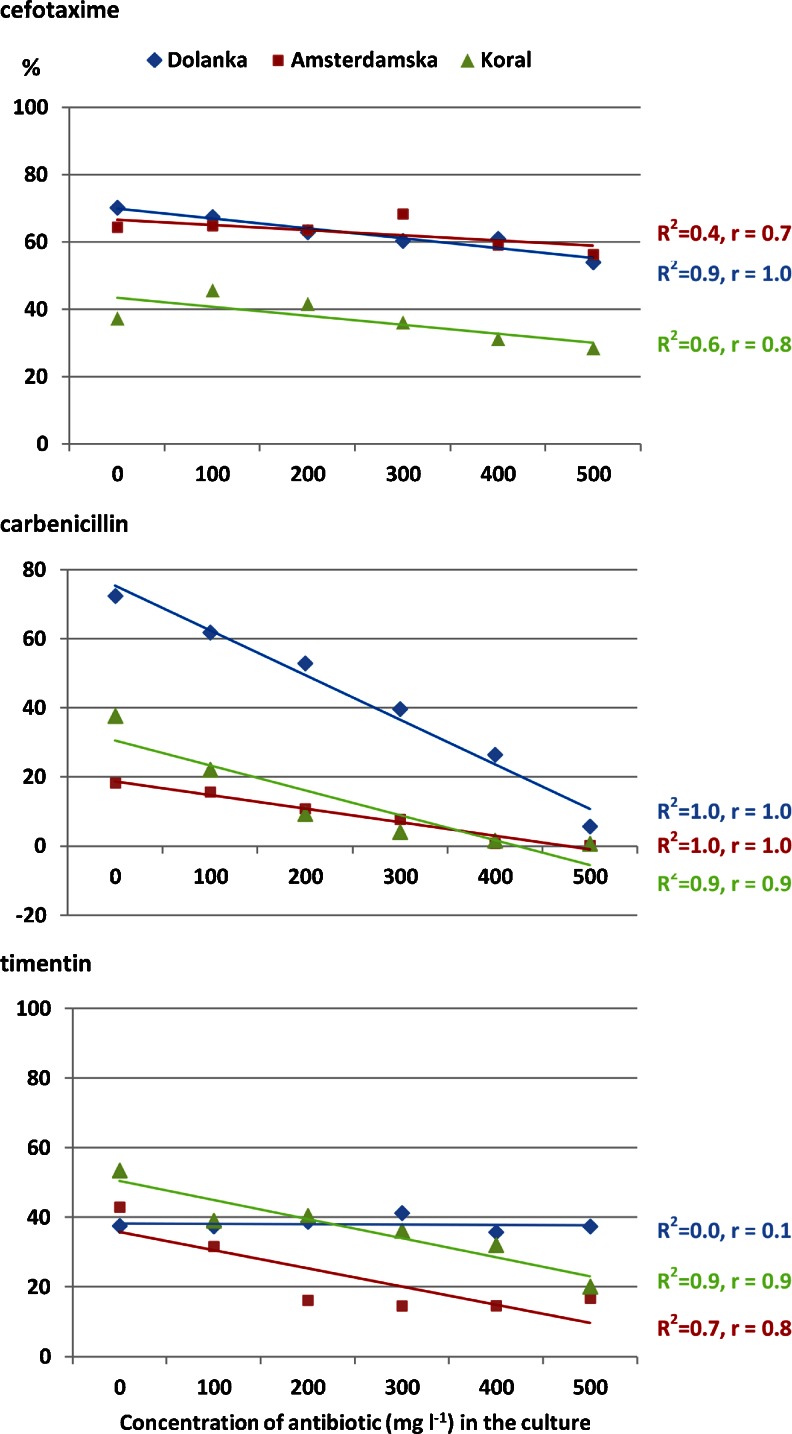
Plating efficiency in 20-d-old protoplast-derived cultures of three carrot accessions in antibiotic-containing media. *R* ^*2*^ coefficient of determination, *r* Pearson’s correlation coefficient.

Both the type and concentration of antibiotic affected the number of cell colonies with respect to protoplast donor accessions (*P* ≤ .01; Table 2). In general, cefotaxime was associated with the least negative influence on plating efficiency. In such conditions, the number of protoplast-derived cell colonies ranged from 37% for ‘Koral’ to 63% for ‘Dolanka’ and ‘Amsterdamska’. The most toxic effects on mitotic divisions were observed after exposing ‘Amsterdamska’-derived protoplasts to carbenicillin and timentin and ‘Koral’-derived protoplasts to carbenicillin (Table 2). In cefotaxime-containing medium, at a concentration of 0–500 mg L^−1^, differences in the level of colony formation were not significant (*P* = .63). In carbenicillin- and timentin-containing media, concentrations of antibiotics higher than 300 and 400 mg L^−1^, respectively, resulted in reductions in plating efficiency in comparison to antibiotic-free medium. A very strong linear reduction in plating efficiency with increasing of antibiotic concentration was recorded for all accessions in carbenicillin-containing media as well for ‘Dolanka’ in cefotaxime-containing medium and for ‘Amsterdamska’ and ‘Koral’ in timentin-containing medium (*R* ^2^ = 0.7–1.0, *P* < .05; Fig. 1). A similar tendency, but marginally non-significant (*P* = .07), was observed for ‘Koral’ in cefotaxime-containing medium.

### Plant regeneration from antibiotic-treated protoplast cultures.

**Figure 2.**
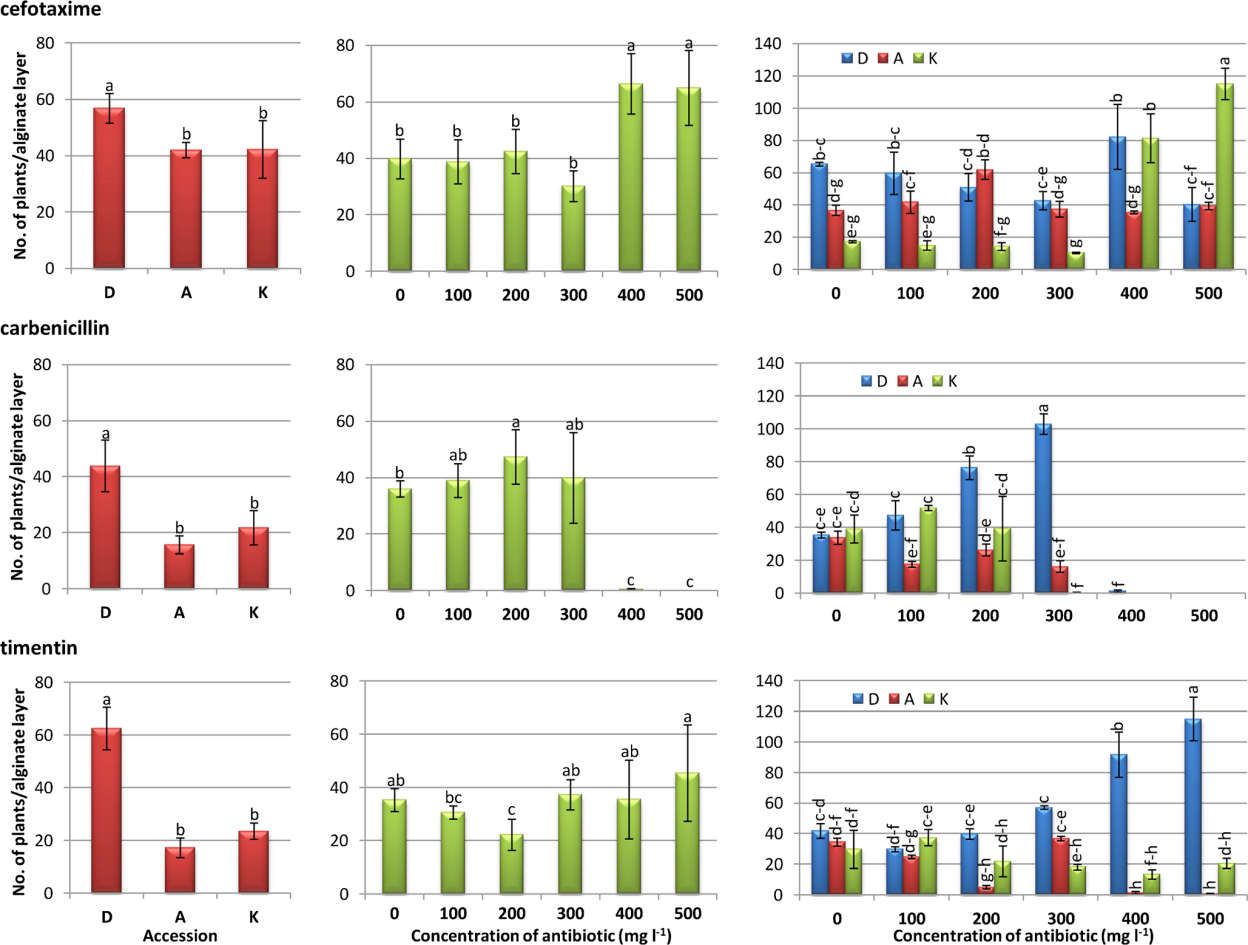
Effect of β-lactam antibiotics on plant regeneration of different carrot accessions from cefotaxime-, carbenicillin-, and timentin-containing protoplast culture media. *Bars* represent the standard error. *D* ‘Dolanka’, *A* ‘Amsterdamska’, *K* ‘Koral’. Means denoted by *different letters* are significantly different (*P* ≤ .001).

During 2 mo of culture in antibiotic-free media, continuous growth of cell colonies in alginate layers took place leading to the formation of microcalli, macrocalli, and proembryonic masses (PEM) in all accessions. PEM easily transformed in sequence into globular, torpedo-shaped, and cotyledonary-stage somatic embryos. On antibiotic-containing media, efficiency of callus and embryo formation varied among accessions, antibiotic type, and concentration (data not shown). Plant regeneration occurred after depolymerization of alginate matrix and transfer of released tissue masses onto hormone- and antibiotic-free media. Similar to calli and PEM development, the number of regenerated plants highly depended on protoplast donor accession and type of antibiotic used during protoplast culture (*P* < .001). On average, the highest number of plants (54.4 ± 4.5) was achieved from ‘Dolanka’-derived protoplast cultures, while almost twofold fewer plants were regenerated from ‘Koral’- and ‘Amsterdamska’-derived protoplast cultures (29.2 ± 4.2 and 24.9 ± 2.5, respectively; Table 3). Such a trend in regeneration efficiency from donor accessions was observed for cefotaxime-, carbenicillin-, and timentin-containing protoplast cultures (*P* < .001; Fig. 2). The production of plants was strongly affected by antibiotic treatment during protoplast cultures reaching on average the highest number from cefotaxime-containing protoplast cultures (47.1 ± 4.0; Table 3) and the lowest from carbenicillin-containing protoplast cultures (27.1 ± 4.1). Various concentrations of antibiotics applied to protoplast cultures differentially influenced plant production (Fig. 2). Exposure of protoplast cultures to 400–500 mg L^−1^ cefotaxime showed a stimulatory effect on plant production in comparison to the control combination (65–66 plants and 40 plants, respectively). On the other hand, 400–500 mg L^−1^ carbenicillin applied to protoplast cultures completely reduced the ability of protoplast-derived tissues to regenerate. However, exposure of protoplast cultures to 200 mg L^−1^ carbenicillin resulted in more plants in comparison to carbenicillin-free protoplast cultures (47 and 36 plants, respectively). For timentin-containing protoplast cultures, a decrease in plant production was observed at a concentration of 200 mg L^−1^, while all other concentrations did not influence the regeneration efficiency (Fig. 2).

**Table 3:** Mean effect of donor accession, antibiotic, and concentration of antibiotic on plant regeneration from antibiotic-treated protoplast cultures

Factor	Number of plants (±SE)/alginate layer
Accession	
Dolanka	54.4 ± 4.5 a
Amsterdamska	24.9 ± 2.5 b
Koral	29.2 ± 4.2 b
Antibiotic	
Cefotaxime	47.1 ± 4.0 a
Carbenicillin	27.1 ± 4.1 c
Timentin	34.3 ± 4.1 b
Concentration of antibiotic (mg L^−1^)	
0	37.0 ± 2.8 a
100	36.1 ± 3.3 a
200	37.3 ± 4.9 a
300	35.7 ± 5.8 a
400	34.1 ± 7.9 a
500	36.8 ± 9.0 a

Means followed by the same *letters* did not differ significantly (*P* ≤ .05)

Strong associations between donor accession, type of antibiotic, and their concentration applied during protoplast culture on plant regeneration were recorded (*P* < .001). For cefotaxime-containing protoplast cultures, an increase in plant production was observed at a concentration of 400–500 mg L^−1^ in ‘Koral’-derived protoplast cultures, while for ‘Dolanka’ and ‘Amsterdamska’ application of cefotaxime to protoplast cultures showed no effect on subsequent plant regeneration (Fig. 2). Carbenicillin present in protoplast culture media at concentrations of 400–500 and 300–500 mg L^−1^ negatively affected plant regeneration for ‘Dolanka’/’Amsterdamska’ and ‘Koral’, respectively. However, a higher regeneration efficiency was recorded from ‘Dolanka’-derived protoplast cultures containing 200–300 mg L^−1^ carbenicillin in comparison to the controls. Timentin applied to ‘Dolanka’- and ‘Amsterdamska’-derived protoplast cultures at concentrations of 400–500 mg L^−1^ stimulated and reduced plant production, respectively, while supplementation of ‘Koral’-derived protoplast cultures with timentin had no effect on plant regeneration.

## Discussion

Despite bacteriostatic (suppression of bacteria growth) and bactericidal (killing of bacteria) effects, antibiotics may behave as plant growth regulators and positively or negatively affect callus induction (Qin *et al.* 2011), plant morphogenesis (Qin *et al.* 2011), shoot formation (Dai and Castillo 2007), somatic embryogenesis (Mittal *et al.* 2009), or root branching (Rahman *et al.* 2004). Generally, β-lactams are considered to be non-toxic to plant cells due to their specific action on bacterial cell walls (Ogawa and Mii 2004), but in some cases, their breakdown products in the culture medium can differently influence plant cell growth; thus, the phytotoxicity of antibiotics can vary markedly between plant species and depends on their concentrations (Tang *et al.* 2000). The present study was the first attempt to assess the effect of three of the most commonly used β-lactam antibiotics: cefotaxime, carbenicillin, and timentin on the regeneration capacity of carrot protoplasts.

A stimulatory effect of cefotaxime in plant tissue cultures was shown, among others, for the following: morphogenesis in maize callus cultures (Danilova and Dolgikh 2004), shoot multiplication and elongation in sugarcane (Kaur *et al.* 2008), somatic embryo formation from callus tissue of indica rice (Grewal *et al.* 2006) and sugarcane (Mittal *et al.* 2009), or more recently on microspore embryogenesis in wheat and triticale (Asif *et al.* 2013). The activity of cefotaxime in the culture may be attributed to the fact that plant esterases degrade it to produce new metabolites that may have growth regulatory properties (Mathias and Boyd 1986). Since a reduced number of albino shoots was observed on cefotaxime-containing media, it has been speculated that cefotaxime can act at the level of chlorophyll synthesis and, thus, boost the photosynthetic machinery (Grewal *et al.* 2006). In addition to this, cefotaxime might inhibit ethylene production in the cultures, which is positively correlated with plantlet differentiation from the callus mass (Pius *et al.* 1993). In contrast, results presented here showed a neutral effect of cefotaxime at all tested concentrations during the early stages of culture (2 wk old) since the mitotic activity of carrot protoplast-derived cells was similar to that observed in control cultures. Both Pollock *et al.* (1983) and Simmonds and Grainger (1993) analyzed the plating efficiency in older 4-wk-old protoplast cultures of *N. plumbaginifolia* and *T. aestivum*, respectively, and concluded that cefotaxime was not toxic up to levels of 100 mg L^−1^. However, during further stages of development (*i.e.*, in 4- to 8-wk-old cultures) the presence of cefotaxime at higher concentrations could positively influence somatic embryogenesis, which was reflected here in the higher number of plants produced from proembryogenic masses derived from carrot protoplast cultures supplemented with 400–500 mg L^−1^ cefotaxime. Similarly, application of 500 mg L^−1^ cefotaxime to callus cultures of sugarcane promoted somatic embryogenesis and subsequent plant regeneration (Mittal *et al.* 2009).

Carbenicillin exhibited dual (stimulatory and inhibitory) impacts on different plant explants (Qin *et al.* 2011). This is because carbenicillin possesses an auxin-like structure, and in culture media, it is broken down to physiologically active auxin phenylacetic acid at levels sufficient to induce auxin-mediated responses (Holford and Newbury 1992). In somatic embryo cultures of walnut, carbenicillin at 100–1,000 mg L^−1^ slightly reduced the production of secondary somatic embryos (Tang *et al.* 2000). Very little growth of callus tissue from root explants of carrots in the presence of 300 mg L^−1^ carbenicillin in the culture medium was also reported (Chang and Schmidt 1991). Our results demonstrated that in early cultures, carbenicillin at concentrations of 400–500 mg L^−1^ reduced the mitotic activity of carrot protoplast-derived cells gradually leading to complete arrest of cell divisions in older cultures, and as a result, a lack of plant regeneration was observed. Yu and Wei (2008) showed that application of carbenicillin to the culture media even at a concentration of 100 mg L^−1^ strongly inhibited plant regeneration from the embryogenic calli of wheat. However, in leaf cultures of horseradish, carbenicillin appeared as a growth enhancer promoting regeneration of adventitious shoots at a concentration of 100 mg L^−1^ and the formation of somatic embryos at concentrations up to 500 mg L^−1^ (Shehata *et al.* 2010). Similarly, in the present research, a positive effect of carbenicillin application to the protoplast cultures at a concentration of 200 mg L^−1^ on regeneration was observed and more plants in comparison with control was produced.

Timentin is one of the novel β-lactams developed recently to enhance antibacterial activity (Demain and Elander 1999). It is composed of ticarcillin coupled with the β-lactamase inhibitor clavulanic acid. Since ticarcillin, belonging to the penicillin group antibiotics, has a similar chemical structure as that of penicillin G, it is metabolized in a similar fashion as carbenicillin to phenylacetic acid, a naturally occurring weak auxin (Nauerby *et al.* 1997; da Silva Mendes *et al.* 2009). Thus, in addition to its broad-spectrum antimicrobial activity, timentin may differentially affect the growth and development of plant explants. The enhancement of organogenesis has been observed on leaf explants of *N. tabacum* (Nauerby *et al.* 1997) and cotyledon explants of tomatoes (Costa *et al.* 2000) at concentrations of 150 and 300 mg L^−1^, respectively. The same concentrations of timentin showed beneficial effects on shoot regeneration in epicotyl explant cultures of sweet oranges (da Silva Mendes *et al.* 2009). In walnut cultures (Tang *et al.* 2000), doses lower than 500 mg L^−1^ had a non-detrimental influence on secondary somatic embryogenesis, whereas in cacao cultures (Silva *et al.* 2009), timentin at 300 mg L^−1^ was associated with a reduced production of somatic embryos. Out of five concentrations of timentin compared in the present study, 500 and 200 mg L^−1^ reduced the formation of cell aggregates and plant regeneration from protoplast-derived cells, respectively, while the remaining concentrations did not exhibit any developmental effects.

Occasional contaminations are most often introduced to cultures randomly by the operator and are usually represented by the genus *Staphylococcus* residing preferentially on human skin scales (Trudeau and Fernández-Caldaz 1994). However, these bacterial isolates can be successfully controlled by cefotaxime at a concentration of 100 mg L^−1^ (Asif *et al.* 2013). In *Agrobacterium*-mediated transformation, the suppression and elimination of agrobacteria in plant tissue, to enable the regeneration of transformed explants or cells, can be observed after application of cefotaxime at doses of 250–500 mg L^−1^ (Nauerby *et al.* 1997; da Silva Mendes *et al.* 2009) or timentin at doses of 150–500 mg L^−1^ (Nauerby *et al.* 1997; Cheng *et al.* 1998; Silva *et al.* 2009). These data may suggest that concentrations of cefotaxime and timentin used in this study could also minimize or eliminate both bacterial contaminations and *Agrobacterium tumefaciens* from carrot tissue cultures without inducing a phytotoxic effect.

## Conclusions

To our knowledge, this study presents the first report evaluating the effect of cefotaxime, carbenicillin, and timentin on plant regeneration in carrot protoplast cultures. Supplementation of protoplast culture media with cefotaxime or timentin in the range of 100–500 mg L^−1^ was essentially non-toxic to the cells and enabled further plant regeneration at high efficiency. Thus, we believe that these antibiotics may be routinely used during complex *in vitro* procedures or in valuable or irreplaceable carrot cultures to prevent them against unwanted and accidental bacterial contaminations. Additionally, cefotaxime and timentin can also be antibiotics of choice to control *Agrobacterium* growth in experiments on genetic transformation of carrots since they exhibit non-detrimental effects on somatic embryogenesis and plant regeneration in protoplast cultures.
